# Supplementary material for: Genome-Wide Association Study of Starch Properties in Local Thai Rice
Source: Plants (Basel). 2023 Sep 17;12(18):3290. doi: 10.3390/plants12183290 (PMC10535886; doi:10.3390/plants12183290)
Supplement: Supplementary file 1 [file plants-12-03290-s001.zip › 1_Supplementary Figure S1_S3.pdf]

A

B

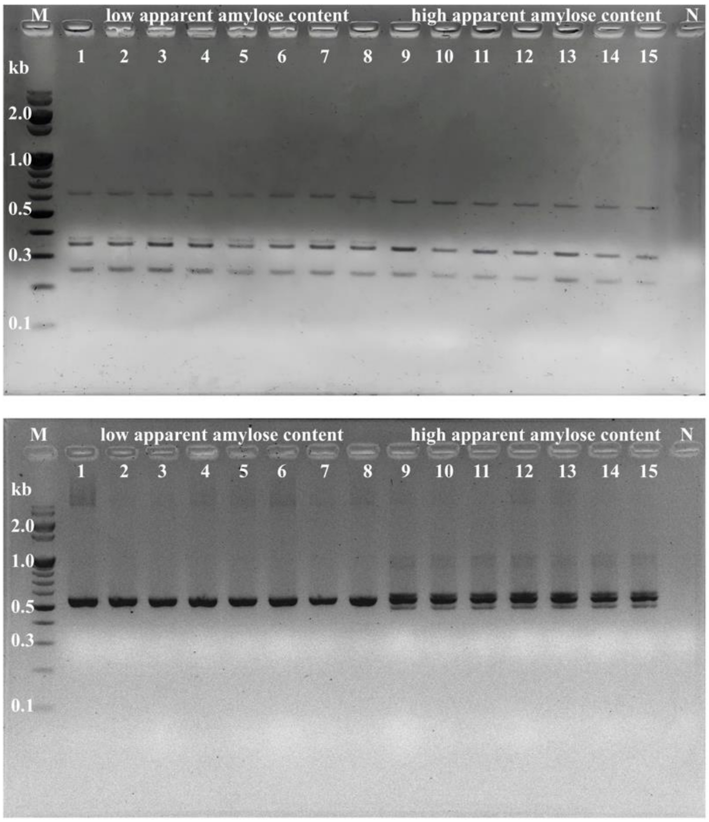

Figure S1. Banding pattern of product from Marker I with BpuEI digestion (A); and banding pattern of product from Marker II (B). Lanes 1-8: PCR amplification of rice with low AAC; Lane 9-15: PCR amplification of rice with high AAC; M: 100 bp molecular weight marker and N: negative control.

```
   reference                MAILLGDVLAPLPIALNLEYAAAIATPPDPALLGRRYHLRRCAAARQDDEEEGEQEQEG-    59
1  KhaoJampi                MAILLGDVLAPLPIALNLEYAATIAMPPDTALLGRRYHLRRRAAARKMMKKKATETMLEK    60
2  NguangChahng             MAILLGDVLAPLPIALNLEYAATIAMPPDTALLGRRHHLRRRAAARKMMKKKATETMLEK    60
3  TahKhui                  MAILLGDVLAPLPIALNLEYAATIAMPPDTALLGRRYHLRRRAAARKMMKKKATETMLEK    60
4  KhaoGawDiaw              MAILLGDVLAPLPIALNLEYAATIAMPPDTALLGRRYHLRRRAAARKMMKKKATETMLEK    60
5  HawmSurin                MAILLGDVLAPLPIALNLEYTATIAMPPDTALLGRRHHLRRRAAARKMMKKKATETMLEK    60
6  JekChuey                 MAILLGDVLAPLPIALNLEYTAAIATPPDPALLGRRYHLRRCAAARQDDEEEGEQEQEG-    59
7  LaiMaejo                 MAILLGDVLAPLPIALNLEYTAAIATPPDPALLGRRYHLRRCAAARQDDEEEGEQEQEG-    59
8  NiawKhiawNgoo            MAILLGDVLAPLPIALNLEYTAAIATPPDPALLGRRYHLRRCAAARQDDEEEGEQEQEG-    59
9  NiawDamHawmPooKhiaw      MAILLGDVLAPLPIALNLEYAAAIATPPDPALLGRRYHLRRCAAARQDDEEEGEQEQEG-    59
10 LawdGwian                MAILLGDVLAPLPIALNLEYTAAIATPPDPALLGRRYHLRRCAAARQDDEEEGEQEQEG-    59

   reference                -HG--NYVGKSTPKVPQLVNELQNRPLTAKPDIQRPLTNQNRSQMVLPWFSPRFCPMWRL    116
1  KhaoJampi                VHRRSLNLSTSYKIVP*-------------------------------------------    76
2  NguangChahng             VHRRSLNLSSSYKIVPQPQNQISNVP*---------------------------------    86
3  TahKhui                  VHRRSLNLSSSYKIVPQPQNQISNVP*---------------------------------    86
4  KhaoGawDiaw              VHRRSLNLSTSYKIVP*-------------------------------------------    76
5  HawmSurin                VHRRSLNLSSSYKIVPQPQNQISNVP*---------------------------------    86
6  JekChuey                 -HG--NYVRKSTSKVPQLVIELQNRPPTAKPDIQRPLTNQNRSQMVLPWFSPRFCPM*--    113
7  LaiMaejo                 -HG--NYVRKSTSKVPQLVIELQNRPPTAKPDIQRPLTNQNRSQMVVPWF*---------    106
8  NiawKhiawNgoo            -HG--NYVRKSTSKVPQLVIELQNRPPTAKPDIQRPLTNQNRSQMVVPWF*---------    106
9  NiawDamHawmPooKhiaw      -HG--NYVRKSTSKVPQLVNELQNRPLTAKPDIQRPLTNQNRSQMVLPWFSPRFCPM*--    113
10 LawdGwian                -HG--NYVRKSTSKVPQLVIELQNRPPTAKPDIQRPLTNQNRSQMVVPWF*---------    106

   reference                SQRGTHVDSIYQAATSTLNTMISTAITRCGRYLQRRSRHNAPDSYLRSSSATRTISLSSA    176
1  KhaoJampi                ------------------------------------------------------------    76
2  NguangChahng             ------------------------------------------------------------    86
3  TahKhui                  ------------------------------------------------------------    86
4  KhaoGawDiaw              ------------------------------------------------------------    76
5  HawmSurin                ------------------------------------------------------------    86
6  JekChuey                 ------------------------------------------------------------    113
7  LaiMaejo                 ------------------------------------------------------------    106
8  NiawKhiawNgoo            ------------------------------------------------------------    106
9  NiawDamHawmPooKhiaw      ------------------------------------------------------------    113
10 LawdGwian                ------------------------------------------------------------    106

   reference                STSAVAAPALRMRHSKSRASLSLPRSVKLLGVSGMDAAPTMTMTAETMSAPSESGSGGET    236
1  KhaoJampi                ------------------------------------------------------------    76
2  NguangChahng             ------------------------------------------------------------    86
3  TahKhui                  ------------------------------------------------------------    86
4  KhaoGawDiaw              ------------------------------------------------------------    76
5  HawmSurin                ------------------------------------------------------------    86
6  JekChuey                 ------------------------------------------------------------    113
7  LaiMaejo                 ------------------------------------------------------------    106
8  NiawKhiawNgoo            ------------------------------------------------------------    106
9  NiawDamHawmPooKhiaw      ------------------------------------------------------------    113
10 LawdGwian                ------------------------------------------------------------    106

   reference                TTTLLGFGVGGDGSQRRRCSA* 257
1  KhaoJampi                ---------------------- 76
2  NguangChahng             ---------------------- 86
3  TahKhui                  ---------------------- 86
4  KhaoGawDiaw              ---------------------- 76
5  HawmSurin                ---------------------- 86
6  JekChuey                 ---------------------- 113
7  LaiMaejo                 ---------------------- 106
8  NiawKhiawNgoo            ---------------------- 106
9 NiawDamHawmPooKhiaw       ---------------------- 113
10 LawdGwian                ---------------------- 106
```

Figure S2. Sequence alignment of *LOC_Os01g65810* in 10 rice cultivars with 5 high AAC (row 1-5) and 5 low ACC (row 6-10); *Nipponbare rice was used as a* r*eference genome sequence.*

```
   reference                 MSALTTSQLATSATGFGIADRSAPSSLLRHGFQGLKPRSPAGGDATSLSVTTSARATPKQ    60
1  KhaoJampi                 MSALTTSQLATSATGFGIADRSAPSSLLRHGFQGLKPRSPAGGDATSLSVTTSARATPKQ    60
2  NguangChahng              MSALTTSQLATSATGFGIADRSAPSSLLRHGFQGLKPRSPAGGDATSLSVTTSARATPKQ    60
3  TahKhui                   MSALTTSQLATSATGFGIADRSAPSSLLRHGFQGLKPRSPAGGDATSLSVTTSARATPKQ    60
4  KhaoGawDiaw               MSALTTSQLATSATGFGIADRSAPSSLLRHGFQGLKPRSPAGGDATSLSVTTSARATPKQ    60
5  HawmSurin                 MSALTTSQLATSATGFGIADRSAPSSLLRHGFQGLKPRSPAGGDATSLSVTTSARATPKQ    60
6  JekChuey                  MSALTTSQLATSATGFGIADRSAPSSLLRHGFQGLKPTGSRASSPAAPPAATRRRSA*--    57
7  LaiMaejo                  MSALTTSQLATSATGFGIADRSAPSSLLRHGFQGLKPTGSRASSPAAPPAATRRRSA*--    57
8  NiawKhiawNgoo             MSALTTSQLATSATGFGIADRSAPSSLLRHGFQGLKPTGSRASSPAAPPAATRRRSA*--    57
9  NiawDamHawmPooKhiaw       MSALTTSQLATSATGFGIADRSAPSSLLRHGFQGLKPTGSRASSPAAPPAATRRRSA*--    57
10 LawdGwian                 MSALTTSQLATSATGFGIADRSAPSSLLRHGFQGLKPTGSRASSPAAPPAATRRRSA*--    57

   reference                 QRSVQRGSRRFPSVVVYATGAGMNVVFVGAEMAPWSKTGGLGDVLGGLPPAMAANGHRVM    120
1  KhaoJampi                 QRSVQRGSRRFPSVVVYATGAGMNVVFVGAEMAPWSKTGGLGDVLGGLPPAMAANGHRVM    120
2  NguangChahng              QRSVQRGSRRFPSVVVYATGAGMNVVFVGAEMAPWSKTGGLGDVLGGLPPAMAANGHRVM    120
3  TahKhui                   QRSVQRGSRRFPSVVVYATGAGMNVVFVGAEMAPWSKTGGLGDVLGGLPPAMAANGHRVM    120
4  KhaoGawDiaw               QRSVQRGSRRFPSVVVYATGAGMNVVFVGAEMAPWSKTGGLGDVLGGLPPAMAANGHRVM    120
5  HawmSurin                 QRSVQRGSRRFPSVVVYATGAGMNVVFVGAEMAPWSKTGGLGDVLGGLPPAMAANGHRVM    120
6  JekChuey                  ------------------------------------------------------------    57
7  LaiMaejo                  ------------------------------------------------------------    57
8  NiawKhiawNgoo             ------------------------------------------------------------    57
9  NiawDamHawmPooKhiaw       ------------------------------------------------------------    57
10 LawdGwian                 ------------------------------------------------------------    57

   reference                 VISPRYDQYKDAWDTSVVAEIKVADRYERVRFFHCYKRGVDRVFIDHPSFLEKVWGKTGE    180
1  KhaoJampi                 VISPRYDQYKDAWDTSVVAEIKVADRYERVRFFHCYKRGVDRVFIDHPSFLEKVWGKTGE    180
2  NguangChahng              VISPRYDQYKDAWDTSVVAEIKVADRYERVRFFHCYKRGVDRVFIDHPSFLEKVWGKTGE    180
3  TahKhui                   VISPRYDQYKDAWDTSVVAEIKVADRYERVRFFHCYKRGVDRVFIDHPSFLEKVWGKTGE    180
4  KhaoGawDiaw               VISPRYDQYKDAWDTSVVAEIKVADRYERVRFFHCYKRGVDRVFIDHPSFLEKVWGKTGE    180
5  HawmSurin                 VISPRYDQYKDAWDTSVVAEIKVADRYERVRFFHCYKRGVDRVFIDHPSFLEKVWGKTGE    180
6  JekChuey                  ------------------------------------------------------------    57
7  LaiMaejo                  ------------------------------------------------------------    57
8  NiawKhiawNgoo             ------------------------------------------------------------    57
9  NiawDamHawmPooKhiaw       ------------------------------------------------------------    57
10 LawdGwian                 ------------------------------------------------------------    57

   reference                 KIYGPDTGVDYKDNQMRFSLLCQAALEAPRILNLNNNPYFKGTYGEDVVFVCNDWHTGPL    240
1  KhaoJampi                 KIYGPDTGVDYKDNQMRFSLLCQAALEAPRILNLNNNPYFKGTYGEDVVFVCNDWHTGPL    240
2  NguangChahng              KIYGPDTGVDYKDNQMRFSLLCQAALEAPRILNLNNNPYFKGTYGEDVVFVCNDWHTGPL    240
3  TahKhui                   KIYGPDTGVDYKDNQMRFSLLCQAALEAPRILNLNNNPYFKGTYGEDVVFVCNDWHTGPL    240
4  KhaoGawDiaw               KIYGPDTGVDYKDNQMRFSLLCQAALEAPRILNLNNNPYFKGTYGEDVVFVCNDWHTGPL    240
5  HawmSurin                 KIYGPDTGVDYKDNQMRFSLLCQAALEAPRILNLNNNPYFKGTYGEDVVFVCNDWHTGPL    240
6  JekChuey                  ------------------------------------------------------------    57
7  LaiMaejo                  ------------------------------------------------------------    57
8  NiawKhiawNgoo             ------------------------------------------------------------    57
9  NiawDamHawmPooKhiaw       ------------------------------------------------------------    57
10 LawdGwian                 ------------------------------------------------------------    57

   reference                 ASYLKNNYQPNGIYRNAKVAFCIHNISYQGRFAFEDYPELNLSERFRSSFDFIDGYDTPV    300
1  KhaoJampi                 ASYLKNNYQPNGIYRNAKVAFCIHNISYQGRFAFEDYPELNLSERFRSSFDFIDGYDTPV    300
2  NguangChahng              ASYLKNNYQPNGIYRNAKVAFCIHNISYQGRFAFEDYPELNLSERFRSSFDFIDGYDTPV    300
3  TahKhui                   ASYLKNNYQPNGIYRNAKVAFCIHNISYQGRFAFEDYPELNLSERFRSSFDFIDGYDTPV    300
4  KhaoGawDiaw               ASYLKNNYQPNGIYRNAKVAFCIHNISYQGRFAFEDYPELNLSERFRSSFDFIDGYDTPV    300
5  HawmSurin                 ASYLKNNYQPNGIYRNAKVAFCIHNISYQGRFAFEDYPELNLSERFRSSFDFIDGYDTPV    300
6  JekChuey                  ------------------------------------------------------------    57
7  LaiMaejo                  ------------------------------------------------------------    57
8  NiawKhiawNgoo             ------------------------------------------------------------    57
9  NiawDamHawmPooKhiaw       ------------------------------------------------------------    57
10 LawdGwian                 ------------------------------------------------------------    57

   reference                 EGRKINWMKAGILEADRVLTVSPYYAEELISGIARGCELDNIMRLTGITGIVNGMDVSEW    360
1  KhaoJampi                 EGRKINWMKAGILEADRVLTVSPYYAEELISGIARGCELDNIMRLTGITGIVNGMDVSEW    360
2  NguangChahng              EGRKINWMKAGILEADRVLTVSPYYAEELISGIARGCELDNIMRLTGITGIVNGMDVSEW    360
3  TahKhui                   EGRKINWMKAGILEADRVLTVSPYYAEELISGIARGCELDNIMRLTGITGIVNGMDVSEW    360
4  KhaoGawDiaw               EGRKINWMKAGILEADRVLTVSPYYAEELISGIARGCELDNIMRLTGITGIVNGMDVSEW    360
5  HawmSurin                 EGRKINWMKAGILEADRVLTVSPYYAEELISGIARGCELDNIMRLTGITGIVNGMDVSEW    360
6  JekChuey                  ------------------------------------------------------------    57
7  LaiMaejo                  ------------------------------------------------------------    57
8  NiawKhiawNgoo             ------------------------------------------------------------    57
9  NiawDamHawmPooKhiaw       ------------------------------------------------------------    57
10 LawdGwian                 ------------------------------------------------------------    57
```

```
   reference                DPSKDKYITAKYDATTAIEAKALNKEALQAEAGLPVDRKIPLIAFIGRLEEQKGPDVMAA    420
1  KhaoJampi                DPSKDKYITAKYDATTAIEAKALNKEALQAEAGLPVDRKIPLIAFIGRLEEQKGSDVMAA    420
2  NguangChahng             DPSKDKYITAKYDATTAIEAKALNKEALQAEAGLPVDRKIPLIAFIGRLEEQKGSDVMAA    420
3  TahKhui                  DPSKDKYITAKYDATTAIEAKALNKEALQAEAGLPVDRKIPLIAFIGRLEEQKGSDVMAA    420
4  KhaoGawDiaw              DPSKDKYITAKYDATTAIEAKALNKEALQAEAGLPVDRKIPLIAFIGRLEEQKGSDVMAA    420
5  HawmSurin                DPSKDKYITAKYDATTAIEAKALNKEALQAEAGLPVDRKIPLIAFIGRLEEQKGSDVMAA    420
6  JekChuey                 ------------------------------------------------------------    57
7  LaiMaejo                 ------------------------------------------------------------    57
8  NiawKhiawNgoo            ------------------------------------------------------------    57
9  NiawDamHawmPooKhiaw      ------------------------------------------------------------    57
10 LawdGwian                ------------------------------------------------------------    57

   reference                AIPELMQEDVQIVLLGTGKKKFEKLLKSMEEKYPGKVRAVVKFNAPLAHLIMAGADVLAV    480
1  KhaoJampi                AIPELMQEDVQIVLLGTGKKKFEKLLKSMEEKYPGKVRAVVKFNAPLAHLIMAGADVLAV    480
2  NguangChahng             AIPELMQEDVQIVLLGTGKKKFEKLLKSMEEKYPGKVRAVVKFNAPLAHLIMAGADVLAV    480
3  TahKhui                  AIPELMQEDVQIVLLGTGKKKFEKLLKSMEEKYPGKVRAVVKFNAPLAHLIMAGADVLAV    480
4  KhaoGawDiaw              AIPELMQEDVQIVLLGTGKKKFEKLLKSMEEKYPGKVRAVVKFNAPLAHLIMAGADVLAV    480
5  HawmSurin                AIPELMQEDVQIVLLGTGKKKFEKLLKSMEEKYPGKVRAVVKFNAPLAHLIMAGADVLAV    480
6  JekChuey                 ------------------------------------------------------------    57
7  LaiMaejo                 ------------------------------------------------------------    57
8  NiawKhiawNgoo            ------------------------------------------------------------    57
9  NiawDamHawmPooKhiaw      ------------------------------------------------------------    57
10 LawdGwian                ------------------------------------------------------------    57

   reference                PSRFEPCGLIQLQGMRYGTPCACASTGGLVDTVIEGKTGFHMGRLSVDCKVVEPSDVKKV    540
1  KhaoJampi                PSRFEPCGLIQLQGMRYGTPCACASTGGLVDTVIEGKTGFHMGRLSVDCKVVEPSDVKKV    540
2  NguangChahng             PSRFEPCGLIQLQGMRYGTPCACASTGGLVDTVIEGKTGFHMGRLSVDCKVVEPSDVKKV    540
3  TahKhui                  PSRFEPCGLIQLQGMRYGTPCACASTGGLVDTVIEGKTGFHMGRLSVDCKVVEPSDVKKV    540
4  KhaoGawDiaw              PSRFEPCGLIQLQGMRYGTPCACASTGGLVDTVIEGKTGFHMGRLSVDCKVVEPSDVKKV    540
5  HawmSurin                PSRFEPCGLIQLQGMRYGTPCACASTGGLVDTVIEGKTGFHMGRLSVDCKVVEPSDVKKV    540
6  JekChuey                 ------------------------------------------------------------    57
7  LaiMaejo                 ------------------------------------------------------------    57
8  NiawKhiawNgoo            ------------------------------------------------------------    57
9  NiawDamHawmPooKhiaw      ------------------------------------------------------------    57
10 LawdGwian                ------------------------------------------------------------    57

   reference                AATLKRAIKVVGTPAYEEMVRNCMNQDLSWKGPAKNWENVLLGLGVAGSAPGIEGDEIAP    600
1  KhaoJampi                AATLKRAIKVVGTPAYEEMVRNCMNQDLSWKGPAKNWENVLLGLGVAGSAPGIEGDEIAP    600
2  NguangChahng             AATLKRAIKVVGTPAYEEMVRNCMNQDLSWKGPAKNWENVLLGLGVAGSAPGIEGDEIAP    600
3  TahKhui                  AATLKRAIKVVGTPAYEEMVRNCMNQDLSWKGPAKNWENVLLGLGVAGSAPGIEGDEIAP    600
4  KhaoGawDiaw              AATLKRAIKVVGTPAYEEMVRNCMNQDLSWKGPAKNWENVLLGLGVAGSAPGIEGDEIAP    600
5  HawmSurin                AATLKRAIKVVGTPAYEEMVRNCMNQDLSWKGPAKNWENVLLGLGVAGSAPGIEGDEIAP    600
6  JekChuey                 ------------------------------------------------------------    57
7  LaiMaejo                 ------------------------------------------------------------    57
8  NiawKhiawNgoo            ------------------------------------------------------------    57
9  NiawDamHawmPooKhiaw      ------------------------------------------------------------    57
10 LawdGwian                ------------------------------------------------------------    57

   reference                LAKENVAAP*   609
1  KhaoJampi                LAKENVAAP*   609
2  NguangChahng             LAKENVAAP*   609
3  TahKhui                  LAKENVAAP*   609
4  KhaoGawDiaw              LAKENVAAP*   609
5  HawmSurin                LAKENVAAP*   609
6  JekChuey                 ----------   57
7  LaiMaejo                 ----------   57
8  NiawKhiawNgoo            ----------   57
9  NiawDamHawmPooKhiaw      ----------   57
10 LawdGwian                ----------   57
```

Figure S3 Sequence alignment of *LOC_Os06g04200* in 10 rice cultivars with high AAC (row 1-5) and low ACC (row 6-10); *Nipponbare rice was used as a reference genome sequence.*
